# Review on Recent Advances and Novel Approaches in Milling and Mashing

**DOI:** 10.1111/1541-4337.70239

**Published:** 2025-08-08

**Authors:** Andreas Laus, Martin Zarnkow, Martina Gastl, Fritz Jacob

**Affiliations:** ^1^ Forschungszentrum Weihenstephan für Brau‐ und Lebensmittelqualität, Technische Universität München, Freising‐Weihenstephan Freising Germany

**Keywords:** beer, brewing, enzymes, mashing, milling

## Abstract

Brewing begins with milling of malt or grain to expose the starchy endosperm. The degree of milling affects the surface of the starch granules, which influences gelatinization and the action of enzymes, especially that of β‐amylase. Excessive comminution can lead to lautering problems and undesirable leaching of substances. During mashing, which is the decisive step in beer production, malt components are converted into soluble forms by enzymatic degradation, and long‐chain starch molecules are particularly affected. Enzymes, such as amylases, play a key role in this process, and their activity is influenced by temperature, pH, and ion content. Recent studies have shown that lower mashing temperatures may be favorable for these enzymes in new grain varieties. Innovations in mashing systems, including continuous mashing and combined crushing and mashing processes, aim to save time and energy and increase yield, with hydrodynamic cavitation showing promise for increasing enzyme activity. This review is focused on the impact of milling techniques, mashing conditions, and technological innovations on enzymatic activity and overall brewing efficiency, providing insights into optimizing beer production processes. In this context, the review consolidates current research on the interplay between comminution and mashing, explores its impact on enzymatic efficiency, and assesses emerging integrated technologies. This is essential for advancing both academic research and innovation in industrial brewing, and new insights in milling and mashing processes as well as engineering advancements can be concluded.

## 1 Introduction

The first two steps in brewing beer are the crushing of the malt or unmalted grain, subsequent mashing, and the mixing of water and grist. In the first step, the raw material is crushed, and the starchy endosperm of the grain or malt is exposed to allow gelatinization and subsequent breakdown by enzymatic activity and heat‐induced physical disintegration during the mashing process. Crushing can be performed using various types of milling and comminution equipment. For modern brewers, the most common devices are roller mills with two, four, or six rolls and hammer mills. Depending on the desired degree of grinding, an appropriate process is best suited for the processes and equipment available in the brewery (Miedl‐Appelbee 2018). The degree of comminution affects the subsequent gelatinization of starch granules and their enzymatic breakdown into fermentable sugars because finer comminution increases the available surface area and enhances both gelatinization and enzyme accessibility. Another advantage of increased comminution is the potentially higher degree of fermentation of the wort. Intensive comminution not only provides increased surface areas but also the possibility of splitting starch chains and improved enzyme activity, especially β‐amylase. It has been proven that an intensive grinding process can split the α‐1,6 bonds of starch chains, especially amylopectin, and increase the proportion of unbranched starch chains, and thus more non‐reducing ends are available for β‐amylase (Wu et al. 2018). However, the extensive grinding of malt leads to problems during lautering if a lauter tun is used. This is because fine particles block the filter bed of the spent grain and prevent the wort from draining off. Undesirable substances, such as polyphenols, can also be extracted from the husk (Freeman 2013).

In order to produce a wort suitable for beer from malt and crude cereals, the malt polymers, including carbohydrates, proteins, and cell wall components, must be converted into soluble form with the aid of enzymatic and physical degradation processes (Narziß and Back 2009; Briggs et al. 2004; Back et al. 2008). The decisive factor in these degradation processes is the action of the enzymes present in the malt if no exogenous enzymes are used. These enzymatic degradation processes are classified as proteolysis, cytolysis, and amylolysis. Because each enzyme relevant to brewing has a specific optimal temperature and pH range for its activity, the temperature‐time parameter is the most important factor for controlling enzymatic processes (Narziß et al. 2017).

Barley and barley malt contain small amounts of soluble sugars such as glucose, sucrose, and fructose (Langenaeken et al. 2020). However, most fermentable carbohydrates must be released during amylolysis, that is, during the enzymatic breakdown of starch chains. Starch behavior during mashing depends on various conditions. Starch grains gelatinize at certain temperatures. In addition to temperature, other factors play a role in gelatinization. For brewing, a low gelatinization temperature is desired so that the starch granules are gelatinized as completely as possible and amylose–amylopectin molecules are freely accessible to enzymes. New research has identified the influence of mash thickness on gelatinization temperature. The protein content of the raw material used and the solubility of the substances natively present in the malt also influence the gelatinization temperature and degradability of starch (W.‐W. Yu, Zhai, et al. 2020; C. F. De Schepper and Courtin 2022).

Enzyme activity has also been analyzed in more recent studies. Each enzyme is characterized by an optimal temperature and pH range in which it exhibits the highest activity. The optimal temperature range for amylolytic enzymes is lower than the reported temperatures owing to new cereal varieties. This allows for lower resting temperatures but must be compared with gelatinization temperatures (Henson and Duke 2016a, 2016b).

Owing to the changing gelatinization temperatures of new barley varieties and findings of factors that influence the gelatinization temperature, mashing temperatures and temperature rests must be adjusted to ensure the efficiency of wort preparation in the brewery. Another factor that must be considered when determining the resting temperature to be maintained during mashing is the activity of malt enzymes. In addition to new barley varieties and climatic developments, mash thickness plays a role in enzymatic activity. The protein and polysaccharide content of malt influences the activity and stability of enzymes. Research has shown that high protein content in malt inhibits enzyme activity, whereas high sugar content improves the thermal stability of the enzymes (Henson and Duke 2016b; Henson et al. 2014; W. Yu, Gilbert, et al. 2020). Wort preparation can be improved through adapted mashing processes, such as simple isothermal mashing, in which the gelatinization temperatures and thermal stability of the enzymes can be considered (Laus et al. 2022).

Depending on the mashing procedure, breweries can use up to two vessels for mashing. This could be a mash cooker that thermally damages the cereals and the main mashing vessel, a mash tube that heats the mash to a specific temperature for optimal enzymatic activity. New mashing systems, such as continuous processes, should be considered that can react rapidly and flexibly to new conditions, gelatinization, and optimum enzyme temperatures. A major aspect of brewery engineering development is the implementation of continuous mashing. The reasons for this are the potentially higher utilization times of vessels, lower energy consumption, and increased yield (Håkansson 2018; Mulder 2012; Strobl 2019; Wefing et al. 2020).

Recent studies on the mechanisms of comminution are analyzed in the context of their relevance to integrated processes that combine comminution and mashing. Wet milling combines crushing and mashing by adding a certain amount of water to malt before crushing, thus initiating the mashing process during milling. Another method involves the use of hydrodynamic cavitation. Here, the malt is crushed in water in the mash tube or in the flow‐through. Hydrodynamic cavitation can be induced by a Venturi nozzle or rotor–stator device. Hydrodynamic cavitation improves the enzyme activity during mashing (Strobl 2019; Albanese et al. 2017).

This review aims to examine the impact of various comminution and mashing techniques on enzymatic activity and overall brewing efficiency. In particular, it focuses on recent research on milling mechanisms, particularly in the context of integrated approaches that combine the milling and mashing processes. Current advancements in process technology, along with new insights into starch gelatinization and enzyme stability, are also reviewed. Given this context, the present review synthesizes current research on comminution and mashing interactions, highlights the implications for enzymatic efficiency, and evaluates emerging integrated technologies. This understanding is crucial for both academic research and industrial brewing innovation.

## 2 Factors Affecting the Milling Process

Milling is the first step of beer production. Using different comminution devices, the grains are broken down, exposing the starchy endosperm. If the grains are not properly milled, this affects the entire brewing process, especially the composition of the wort and mashing efficiency. The presence of intact grains or large fragments means that the inner parts of the grain are not exposed, resulting in low conversion of starch into fermentable sugars and low final mashing yields. However, excessive milling leads to the extraction and dissolution of compounds such as polyphenols, which cause undesirable properties in wort and beer (de Moura and dos Santos Mathias 2018).

**FIGURE 1:**
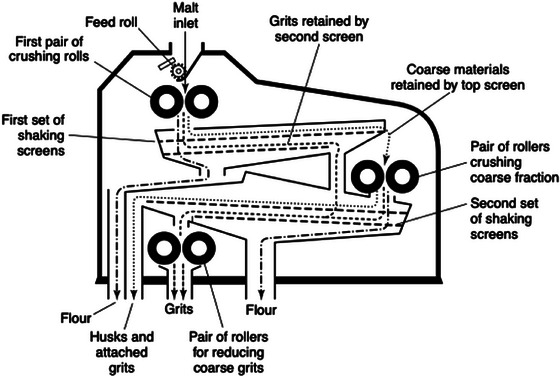
Scheme of a six‐roll mill (Briggs et al. 2004).

**FIGURE 2:**
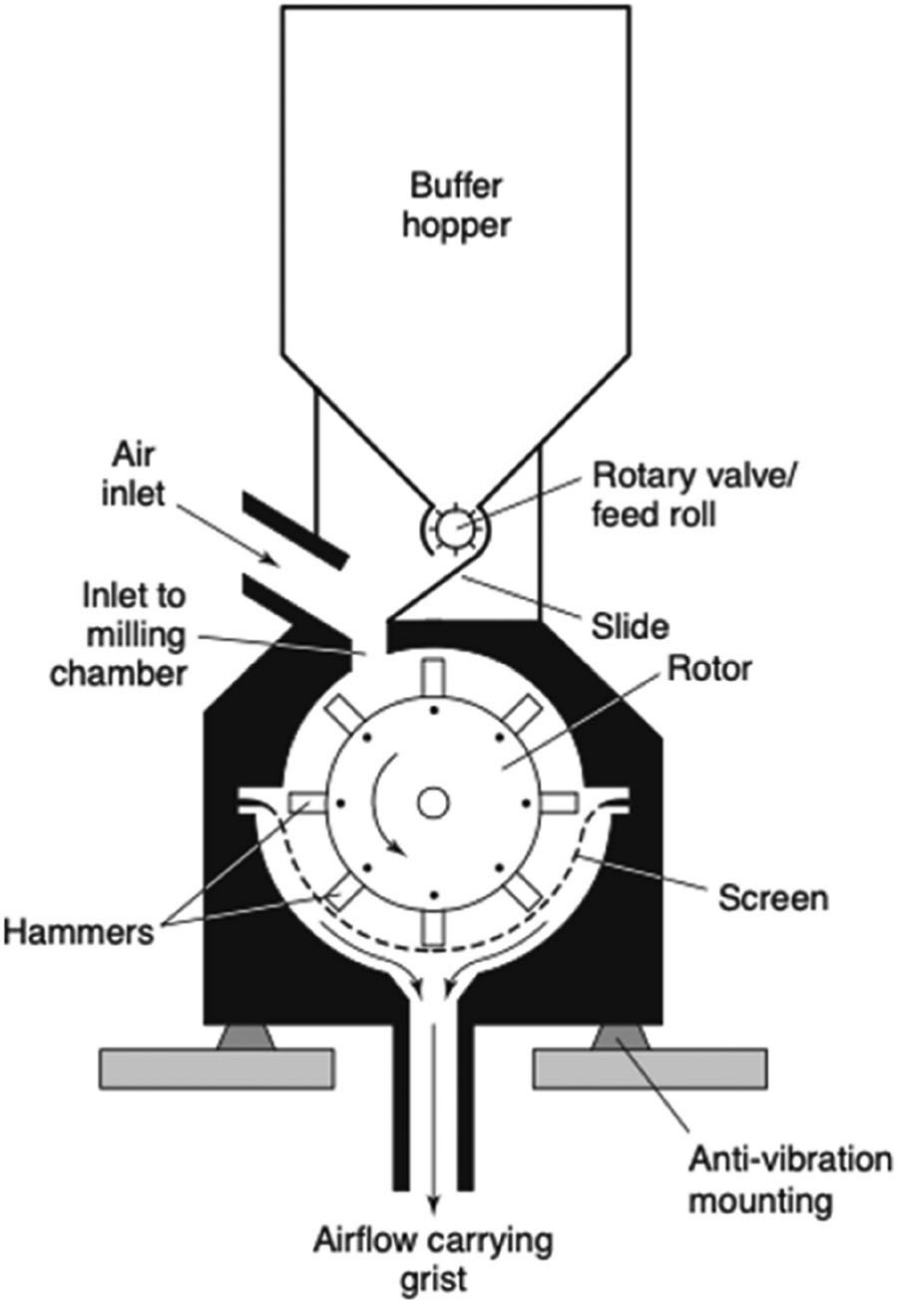
Scheme of a hammer mill (Briggs et al. 2004).

**FIGURE 3:**
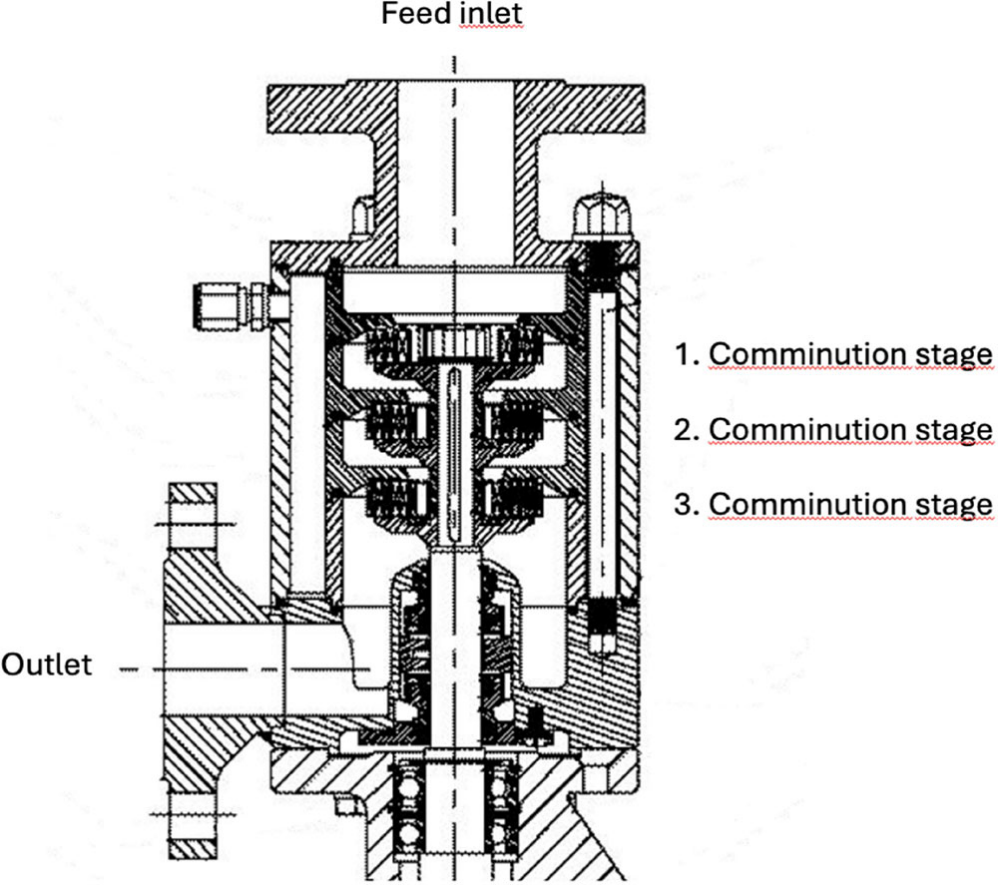
Layout of rotor–stator comminution device (IKA Dispax) (IKA Works).

Substrate milling is classified into various loading mechanisms. These loading mechanisms can be divided into impact, compression, shear, and attrition (Parapari et al. 2020) and are listed in Table 1. For brewing purposes, only the first two principles and, to a lesser extent, the third are relevant. Each comminution machine experiences various loading mechanisms, with one or several typically dominant mechanisms. A compression loading mechanism occurs when a particle is pressed between two surfaces, either between two tools, such as rolls in a mill, or between a tool and part of a comminution machine. Roller mills are common in breweries and occur with two, four, or six rolls. Figure 1 illustrates a six‐roller mill. Impact loading mechanisms are characterized by the rapid application of force. During impact loading, the machine blades or accelerated media, such as those in a hammer mill, hit the particle. This impact transfers momentum, propelling the particle to a speed of up to 160 m/s and allowing it to collide with other particles or the lining of the machine. Hammer mills, as shown in Figure 2, are also common in breweries in combination with a mash filter. Shear is another type of loading in comminution environments. Shear loading results in wear and shear stress. This mechanism occurs in two scenarios: When a particle is trapped between two surfaces or other particles, or when particles collide sideways as they pass each other (Parapari et al. 2020). This mechanism is less common in breweries, but systems such as Dispax exist, as shown in Figure 3.

The crushing or grinding of grains or malt releases starch for enzymatic hydrolysis. During milling, the starch grains are damaged by various shear and pressure forces. These forces influence their morphology and functional properties such as solubility, swelling capacity (SP), heat stability, retrogradation, pasting behavior, and digestibility (Tian et al. 2022; Bangar et al. 2023). For example, strong grinding can be observed in ball mills, which changes the grain morphology, molecular weight, crystallinity, and amylose/amylopectin ratio of different starches (Chorfa et al. 2022; Noor et al. 2021; Tan et al. 2015). Strong grinding and the associated particle size distribution lead to an increased surface area and non‐reducing ends in starch molecules (Chitrakar et al. 2020). In starch with high amylopectin content, intensive grinding leads to a reduction in molecular size and an increase in amylose content. This is due to the mechanical separation of the α‐1,6‐branches of amylopectin, which releases the α‐1,4‐chain branching and is measured as amylose. Amylose content increases with increasing grinding time (Bangar et al. 2023). Mechanically processed starch has a significantly higher amylose content than native starch, and a higher processed starch content leads to a greater increase in amylose at the expense of amylopectin conversion (Wang et al. 2020).

Mechanically processed starch is produced by a grinding process in which starch granules are subjected to various forces such as shear, impact, collision, and/or friction (Suki et al. 2016; K. Zhang, Dai, et al. 2019; Wang et al. 2020). The water absorption capacity and hydrolysis rate of starch changed depending on the degree of comminution of the processed starch. A high degree of grinding produces products that can be easily fermented by yeast. Mechanical disintegration of starch leads to the destruction of its granular structure (Zavareze and Dias 2011). Starch granules become rough, distorted, and deformed owing to mechanical treatment (Sakhare et al. 2014; Wronkowska and Haros 2014). The crystalline regions of starch grains are destroyed by mechanical stress, which leads to a decrease in the degree of crystallinity compared to that of native starch (Loubes and Tolaba 2014; Palavecino et al. 2019). The crystalline region is more susceptible to mechanical forces than the amorphous region and becomes smaller as the starch is damaged (Wang et al. 2020; Kim and Kim 2014). Starch subjected to greater mechanical stress has an improved rate of enzymatic hydrolysis compared to native starch (Dhital et al. 2010; Fu et al. 2016; J. Yu et al. 2015). This is because of the smaller particle size and large surface area, which increase the interaction between the enzyme and substrate (Almeida et al. 2019; Lv et al. 2019; Wang et al. 2020).

A higher proportion of mechanically processed starch leads to a significant reduction in the proportion of longer molecular chains and an increase in the proportion of shorter chains (Wu et al. 2018). The gelatinization temperature decreases when starch is subjected to high mechanical stress because of the increasing disorder in the crystalline structure due to the degradation of amylopectin or amylose, which reduces the energy required for gelatinization (C. Liu et al. 2017; R. Liu et al. 2019; Shi et al. 2016; Wang et al. 2020).

Starches that have been heavily comminuted by ball milling exhibit higher swelling and greater water absorption. Strong grinding damages the matrix structure, enabling water bonding through hydrogen bonds and increasing swelling (R. Punia et al. 2017; S. Punia 2020a, 2020b; Bangar et al. 2023).

However, several disadvantages that depend on the milling device used are observed. Steep conditioning, that is, soaking of malt in water before milling, and a drastic increase in temperature during milling cause high wear on the roller riffles and therefore result in high maintenance costs. The investment costs for roller mills without conditioning are often very high, depending on the desired fractionation and resulting number of rollers. In addition, the throughput may be extremely low. Hammer mills require a high motor output, which means that the energy requirement is very high per ton of cereal. Regular maintenance of the hammers is necessary. In addition, all these systems are designed to process only one or two different types of grains (Miedl‐Appelbee 2018).

Due to the increased use of unmalted grains in conjunction with technical enzymes (Kok et al. 2019), the technical requirements for machines to crush raw materials have increased (Aastrup 2010). Unmalted grains are considerably harder than malted grains (Loiko and Romanova 2018), requiring six‐roller and hammer mills. The wear of crushing tools, such as rollers or hammers, is greatly increased when crushing crude grains and must be considered when using them, as crude grains are less friable (Kok et al. 2019; Steiner et al. 2012). Therefore, new machines and materials for milling raw cereals are necessary to reduce maintenance costs and the duration of the milling process (Kaushal and Kumar 2022). High proportions of unmalted cereals were crushed separately. If a two‐roller mill is used, then the grinding gap should be 0.2 mm (Loiko et al. 2018).

The energy consumption of crushing systems depends not only on the type of system but also on the material to be milled. The energy requirement varies depending on whether the materials are malted, as unmalted cereals are significantly more difficult to mill. For example, the energy requirement of a two‐roller mill is approximately 35 times higher for milling crude barley than for milling barley malt (Chladek et al. 2018). Milling oats is also more energy‐intensive than crushing malt, and hammer mills require more energy than roller mills (Schnitzenbaumer et al. 2012). Wet milling, which involves milling grains soaked in water, and conditioned milling, in which grains are sprayed with water before milling, can reduce the energy requirements of mills (Zogg 1993; Ferstl et al. 2002). In addition to the material to be milled, the roller spacing determines the energy requirements of roller mills. The specific energy per quantity of raw material increased with decreasing roller spacing. The feed rate, on the other hand, plays no role in the energy required, considering a continuous feed. For hammer mills, screen opening size primarily influences the energy consumption. The smaller the opening of the screen, the finer the cereals that need to be milled; therefore, more energy is required (Fang et al. 1997).

## 3 Mashing

### 3.1 Objectives of Mashing

The main task of mashing is to dissolve malt components that are relevant for fermentation and to degrade components, like β‐glucans and long‐chain proteins, that are undesirable in the brewing process. Malt constituents, including carbohydrates, proteins, and cell wall substances, must be converted into soluble form with the aid of enzymatic and physical degradation processes (Narziß and Back 2009; Briggs et al. 2004; Back et al. 2008). Long‐chain starch molecules are degraded into glucose, maltose, and maltotriose (Fox 2017). The decisive factor in these degradation processes is the action of enzymes present in the malt. The enzymatic degradation during mashing was divided into three processes. These processes are proteolysis, the enzymatic breakdown of proteins; cytolysis, the degradation of structural substances such as hemicellulose and gums; and amylolysis, the enzymatic breakdown of starch chains. The enzymes involved in the degradation processes during the mashes are listed in Table 2.

**TABLE 1:** Characteristics of different comminution systems for malt grinding.

Grinding type	Mill type	Throughput (t/h)	Specific energy consumption (kWh/t)
Compression	Two‐roller dry	0.25–2.5 (Manger 2019)	8.2 (Smejtková 2016; Smejtková and Vaculik 2018)
Compression	Two‐roller wet	up to 20 (Manger 2019)	2.5 (Manger 2019)
Compression	Two‐roller conditioned	5.0–25.0 (Manger 2019)	2.9 (Manger 2019)
Compression	Four‐roller dry	0.5–6.0 (Manger 2019)	2.3 (Manger 2019)
Compression	Four‐roller conditioned	up to 40 (Manger 2019)	3.0 (Manger 2019)
Compression	Six‐roller dry	up to 15.0 (Manger 2019)	1.8–2.2 (Manger 2019)
Impact	Hammer mill	up to 20.0 (Schwill‐Miedaner 2011)	7.0–12.0 (Schwill‐Miedaner 2011; Tumuluru 2014)
Impact	Hydro mill	10.8–15.5 (Schwill‐Miedaner 2011)	6.3–6.9 (Schwill‐Miedaner 2011)
Compression/Shear	T‐Rex	6.0 (Schwill‐Miedaner 2011)	4.5 (Schwill‐Miedaner 2011)
Shear	Dispax	up to 30.0 (Schwill‐Miedaner 2011)	5.6 (Narziß and Back 2009)

**TABLE 2:** Characteristics of the enzymatic brewing processes.

	Enzyme	Temperature optima (°C)	Inactivation temperature (°C)	pH optimum range
Cytolysis	β‐glucan‐solubilase	60–65 (Narziß and Back 2009)	73 (Narziß and Back 2009)	5.0–6.5 (Narziß and Back 2009)
	Endo‐1,3‐β‐glucanase	40–45; 60 (Narziß and Back 2009; Briggs et al. 2004)	>50; 70 (Narziß and Back 2009; Briggs et al. 2004)	4.4–5.6 (Narziß and Back 2009; Briggs et al. 2004)
	Endo‐1,4‐β‐glucanase	40–45 (Narziß and Back 2009; Briggs et al. 2004)	55 (Narziß and Back 2009; Briggs et al. 2004)	4.5–4.8 (Narziß and Back 2009; Briggs et al. 2004)
	Endo‐1,3‐1,4‐β‐glucanase	37–55 (Lauer et al. 2017)	65 (Lauer et al. 2017)	4.0–8.0 (Chaari and Chaabouni 2019)
	Exo‐β‐glucanase	40 (Kreisz 2002)	>40 (Kreisz 2002)	4.5 (Kreisz 2002)
	Cellobiase	60 (Tiwari and Verma 2017)	–	5.0 (Tiwari and Verma 2017)
	Laminaribiase	37 (Kreisz 2002)	>55 (Kreisz 2002)	5.0 (Kreisz 2002)
	Endo‐xylanase	40–45 (Malfliet et al. 2010; Peng et al. 2019)	>70; 75 (Kanauchi et al. 2013)	4.6–6.5; 5.5–7.5 (Kanauchi et al. 2013; Peng and Jin 2020)
	Xylosidase	65 (Vanbeneden et al. 2008)	>70 (Vanbeneden et al. 2008)	4.0–5.5 (Kanauchi et al. 2013)
	Arabinofuranosidase	49; 50 (Faltermaier 2015)	>50 (Debyser et al. 1998)	5.9–7.5 (Gastl et al. 2021)
	Feruloyl esterase	25–30 (Humberstone and Briggs 2012)	65 (Vanbeneden et al. 2008)	5.4–6.6 (K.J. Schwarz et al. 2012)
Proteolysis	Endopeptidase	45; 60 (Narziß and Back 2009)	>60 (Narziß and Back 2009)	4.5–5.0 (Kerpes et al. 2017)
	Carboxypeptidase	40–60 (Narziß and Back 2009)	70 (Narziß and Back 2009)	4.8–5.6 (Narziß and Back 2009)
	Aminopeptidase	52 (Oszywa et al. 2013)	>60 (Oszywa et al. 2013)	6.0–9.0 (Oszywa et al. 2013)
	Dipeptidase	40–50 (Narziß and Back 2009)	>50 (Narziß and Back 2009)	7.8–8.2 (Narziß and Back 2009)
Amylolysis	β‐Amylase	55–65 (C. De Schepper and Courtin 2021; C.F. De Schepper et al. 2021; Henson and Duke 2016a)	74 (D.E. Evans and Fox 2017)	5.4–5.6 (Narziß and Back 2009; Briggs et al. 2004)
	α‐Amylase	63–74 (Henson and Duke 2016b)	>80 (C. De Schepper and Courtin 2021; C.F. De Schepper et al. 2021)	5.6–5.8 (Narziß and Back 2009; Briggs et al. 2004)
	Maltase	50–55 (Laus et al. 2022)	>63 (Laus et al. 2022)	4.0–4.5 (Narziß and Back 2009)
	Limit dextrinase	∼65 (D.E. Evans and Fox 2017)	>74 (D.E. Evans and Fox 2017)	5.1 (Narziß and Back 2009)
	Invertase	50–60 (Narziß and Back 2009)	63 (Laus et al. 2022)	5.5 (Narziß and Back 2009)
	α‐glucosidase	<70 (Henson et al. 2018; Im and Henson 2021)	>70 (Henson et al. 2018; Im and Henson 2021)	4.6 (Briggs et al. 2004)
Other enzymes	Lipase	45–60 (P. Schwarz et al. 2002)	>74 (P. Schwarz et al. 2002)	6.8–7.0 (Narziß and Back 2009)
	Lipoxygenase	35 (Sun et al. 2012)	70 (Sun et al. 2012)	6.8 (Sun et al. 2012)
	Phosphatase	50–53 (Preece 1963)	>60 (Preece 1963)	5.0 (Preece 1963)
	Peroxidase	50–65 (Narziß and Back 2009)	>90 (Narziß and Back 2009)	5.0–7.0 (Kanauchi 2017)
	Ascorbic acid oxidase	40 (Kanauchi et al. 2014)	>100 (Kanauchi et al. 2014)	7.0 (Kanauchi et al. 2014)
	Thiol oxidase	–	>65 (Bamforth et al. 2009)	5.0–8.0 (Bamforth et al. 2009)
	Oxalate oxidase	–	>80 (Kanauchi et al. 2009)	>80 (Kanauchi et al. 2009)
	Superoxide dismutase	45–55 (Bamforth 1983, 2001)	>65 (Bamforth 1983, 2001)	∼5.2 (Bamforth 1983, 2001)
	Catalase	—	65 (Bamforth et al. 1993)	—

#### 3.1.1 Starch

Starch is the main component of barley, with a proportion of 51%–77%. Barley and wheat starches had a bimodal distribution of starch granules. Barley starch granules consist of large, lenticular A‐type granules with a diameter of 10–40 µm and small, spherical B‐type granules, which have a diameter of 1–10 µm (C. F. De Schepper, Gielens, et al. 2021; Mello El Halal et al. 2019; Ao and Jane 2007; C. F. De Schepper et al. 2020). The average diameter of the large granules is between 15 and 19 µm, and the diameter of the small granules is between 3.1 and 3.7 µm (Bertoft 2017). The large A‐type granules make up 10%–20% of the total starch granules, with a mass fraction of 90%–95%. Small B‐type granules make up the largest proportion of starch granules at 80%–90%, but only account for 10%–15% of the total starch in the malt by mass (S. Punia 2020b). Although 32%–39% of the starch in raw barley consists of small B‐type granules, this proportion is reduced to 17%–27% during malting. This indicates that during malting, small starch granules are hydrolyzed more quickly than large A‐type starch granules (C. F. De Schepper et al. 2020). In some studies, the smallest B‐type granules were assigned to a separate class, C‐type. The different morphologies of A‐ and B/C‐type starch granules in barley and other cereals are a consequence of their different amylopectin structures (S. Punia 2020b). Type B starch granules were more stable than type A granules. This was due to the presence of hydrogen bonds. Although A‐type granules have only eight interhelical water molecules, B‐type granules have 36. Therefore, more hydrogen bonds can form in B‐type granules (Tan et al. 2015; Wang et al. 2020).

Starch granules can be described based on their structural and hierarchical structures. At the molecular level, starch granules are composed of amylose and amylopectin, which form lamellar and semi‐crystalline structures that lead to concentric growth rings, ultimately defining the granular structure (Liang et al. 2023; Bertoft 2017; Chen et al. 2017). Starch granules consist almost exclusively of two polysaccharides, long‐chain amylose and branched short‐chain amylopectin. Both of these polysaccharides consist of chains of α‐(1,4)‐linked d‐glucose residues, which are interconnected through α‐(1,6)‐glucosidic linkages, thus forming branches in the polymers. In common literature, amylose is usually described as unbranched, but amylose also has some α‐(1,6) branches. Amylose consists of long chains of several hundred or thousand glucose units (Chu et al. 2014). In most naturally occurring starch sources, amylose makes up less than 35%, and, unlike amylopectin, amylose is not necessary for the formation of semi‐crystalline structures. It consists of long linear α‐1,4‐linked chains (typically DP 100–10,000) with rare α‐1,6‐linked branch points and is probably found in amorphous regions of starch granules (Bertoft 2017; S. Punia 2020b). Amylopectin, on the other hand, has many branches and consists of comparatively short glucose chains (Nada et al. 2017). The branches of amylopectin constitute approximately 5% of the molecule, which results in a very complex molecular structure (Bertoft 2017; Quek et al. 2019). Amylopectin forms a semi‐crystalline structure in starch granules. Neighboring amylopectin chains form double helices, which, in turn, join to form crystalline lamellae. Short amylopectin chains form double helices, which crystallize and contribute to the semi‐crystalline nature of the starch granules. Amylose is embedded in a semi‐crystalline matrix of starch granules (Seung 2020; Bertoft 2017). When starch granules are heated in an aqueous suspension, they swell to varying degrees depending on the temperature, and amylose tends to separate from the granules. The gelatinization temperature plays a decisive role in the hydrolysis of starch (Bertoft 2017).

#### 3.1.2 Gelatinization

Gelatinization involves the melting of pseudocrystalline areas of amylopectin, which is accompanied by limited leaching of amylose. This included intensive swelling and complete disintegration of the starch granules. The onset of gelatinization was characterized by an abrupt increase in viscosity. Mashing at the beginning of gelatinization led to limited swelling of the A‐type granules and no swelling of the B‐type granules. Therefore, mashing at this temperature leads to limited hydrolysis (Rittenauer et al. 2021).

Small B‐type starch granules are not gelatinized at a temperature of 62°C. These starch granules gelatinize at temperatures between 62°C and 78°C. A larger proportion of dextrin was released during the hydrolysis of small starch granules than during the hydrolysis of large starch granules. This is due to the reduced activity of β‐amylase at these temperatures (Langenaeken et al. 2019). B‐type starch granules have higher gelatinization temperatures than type A starch granules. In tests by De Schepper et al., this was 62.8°C for B‐type granules and 59.6°C for A‐type granules. Mashing at the pasting temperature, which is on average 2.6°C higher than the onset gelatinization temperature, leads to significant swelling of the A‐type granules and subsequent amylolytic hydrolysis. However, this temperature is not sufficient to completely gelatinize and hydrolyze A‐ and B‐type granules. Increasing this temperature led to complete swelling of the A‐type granules and partial swelling of the B‐type granules after 10 min of mashing (Rittenauer et al. 2021).

The gelatinization temperature is dependent on different factors. In addition to the growth conditions of the grains, malting affects the gelatinization temperature. Malting causes the gelatinization temperature to rise by 1.2°C. This increase was due to a change in the intrinsic properties of starch granules and the formation of water‐soluble non‐starch components in the endosperm during malting. A higher content of water‐soluble components in barley malt leads to higher initial starch gelatinization temperatures, especially when mashed with high malt–water ratios (C. F. De Schepper and Courtin 2023). The presence of water‐soluble non‐starch components in the endosperm of the barley malt increases its gelatinization temperature about 4.6°C. This leads to a gelatinization temperature of over 67°C, which is well above the inactivation temperature of β‐amylases (C. F. De Schepper and Courtin 2022, 2023).

Non‐starch components present in barley malt or formed during malting increase the intrinsic gelatinization temperature of starch granules. During mashing, large starch granules gelatinize first and are quickly hydrolyzed, leading to a rapid increase in sugar and dextrin concentrations. This led to increased gelatinization temperatures for the remaining non‐gelatinized, mainly small, starch granules. Therefore, higher malt quantities in the mash lead to a higher quantity of extracted malt components and, thus, higher starch gelatinization temperatures (C. F. De Schepper and Courtin 2022). The yield of fermentable sugars in the wort decreased as the malt/water ratio increased. Sugar and other components in the mash increase the gelatinization temperature of starch. Increasing the amount of extracted components by 2.5 g per 100 g of wort during mashing increased the starch gelatinization temperature by 1°C for both types of granules. This result suggests that at high mash thicknesses, gelatinization is delayed such that β‐amylase ceases to produce the maximum amount of fermentable sugars, especially from small starch granules (Langenaeken et al. 2019).

During mashing, starch gelatinizes at a certain temperature. Amylose is then washed out into the mash, which slows enzyme diffusion and impairs the enzymatic hydrolysis of starch. In addition, long‐chain amylose molecules can become entangled with amylopectin chains in the crystalline lamellae and co‐crystallize, resulting in limited starch swelling. Owing to this limited swelling, the hydrolysis of starch by malt amylases is limited, resulting in a lower maltose content at the end of mashing. Malt amylose content is negatively correlated to starch hydrolysis during mashing. Barley malt, with predominantly short‐chain amylose molecules, has a higher content of fermentable sugars after mashing. This result is observed because short‐chain amylose is more easily leached out of the granule structure and is freely available for enzymatic hydrolysis, loosening the structure of the remaining starch. This observation may explain the influence of both the amylose content and chain length distribution on the release of fermentable sugars (W. Yu et al. 2019).

Another factor that influences the gelatinization temperature is the malt protein content. The protein content of starch correlates positively with the gelatinization temperature. Higher protein content leads to a higher gelatinization temperature (Fox et al. 2019). The protein and amylose contents in both unmalted and malted barley were significantly negatively correlated with the fermentable sugar content after mashing. Proteins may inhibit the swelling of starch granules, which worsens enzymatic degradation. Barley with higher protein content requires higher mashing temperatures to obtain higher fermentable sugar content at the end of mashing. Barley with lower protein and amylose contents, smaller amylose molecule sizes, and fewer long amylopectin chains releases more fermentable sugars at the end of mashing (W. Yu et al. 2019).

If barley malt with a higher protein content is mashed for 60 min at a temperature of 65°C, then not all starch granules have gelatinized, and small starch granules remain completely undigested. Thus, a higher gelatinization temperature is required. If the malt has a lower protein content and is mashed under the same conditions, then all starch granules are gelatinized, and even small starch granules are broken down to a greater extent. A lower gelatinization temperature is required for lower protein content. The presence of other malt components, especially malt protein, inhibits the gelatinization of malt starch (W.‐W. Yu, Zhai, et al. 2020).

In malts with low β‐amylase activity, the removal of proteins leads to a significantly increased content of fermentable sugars. No significant changes were observed in malts with higher β‐amylase activity. Therefore, the protein content influences the gelatinization temperature and enzymatic activity. Malt proteins influence the efficiency of mashing, especially that of β‐amylase. The presence of protein bodies and other substances surrounding the starch granules reduces water absorption/uptake into the granules, thus hindering starch gelatinization. Therefore, an increased gelatinization temperature is required (W. Yu, Gilbert, et al. 2020). The protein content of barley correlated significantly and negatively with the molecular structure of starch. Lower protein content leads to larger amylose and amylopectin chains and a higher proportion of large A‐type granules. Barley with a lower protein content would have a higher starch content and a larger granule size and would therefore be preferable for brewing. It has been hypothesized that the presence of hordein in barley inhibits the enzymatic hydrolysis of starch granules. Possible reasons for impaired hydrolysis with increased protein content are the physical adsorption of barley protein with starch‐degrading enzymes and the physical hindrance of the enzyme by the barley protein matrix (W. Yu et al. 2017). At a mashing temperature of 45°C, the barley protein content in the mash remains relatively constant. The protein content begins to decrease when the temperature of the mash is heated to 65°C and remains at a constant level when the temperature is increased to 70°C. As proteolytic enzymes are active at lower temperatures, the loss of proteins is due to physical processes, such as precipitation or adsorption. These changes in soluble proteins were due to the physical removal of proteins and not enzymolysis (Aldred et al. 2021).

The gelatinization temperature is negatively correlated to the fermentable sugar content, indicating that β‐amylase is only active to a limited extent in malt with a higher gelatinization temperature, which leads to a lower maltose content. A gelatinization temperature >65°C leads to a lower content of fermentable sugars in the wort owing to insufficient enzyme activity (D. E. Evans 2017; D. E. Evans and Li 2009). When a mashing process started at a temperature of 45°C, well below the gelatinization temperature, amylolytic enzymes are active and able to hydrolyze starch, albeit less efficiently (Fox et al. 2019). The glucose content increases when the temperature rises to 62°C. At this temperature, starch gelatinizes and is solubilized by amylases. At the beginning of the mashing process, the sugars present in the malt enter the solution. This included approximately 5% sucrose, which is dissolved in the mash. During mashing, sucrose is converted into glucose and fructose. This conversion takes place by malt invertases (Vriesekoop et al. 2010; Laus et al. 2022), which explains the decrease in the sucrose content at a temperature of 45°C. At higher temperatures, this reaction occurs chemically in acidic environments (Bower et al. 2008). At 45°C, the glucose, maltose, and maltotriose concentrations in the wort slightly increase. Although the glucose content increases steadily, maltose and maltotriose are formed in large quantities when the temperature rises to 62°C. At these temperatures, the starch gelatinizes, and amylolytic enzymes, especially β‐amylase, produce maltose (D. E. Evans et al. 2003). Once the mash temperature reaches 72°C, the concentration of fermentable sugars remains constant. The soluble starch is only reduced in its degree of polymerization by the action of α‐amylase at these temperatures, and no fermentable sugars are formed (Langenaeken et al. 2020).

#### 3.1.3 Amylolytic Activity During Mashing

As many proteolysis and cytolysis processes already occur during malting, particular attention is paid to the mashing process in the brewery during starch digestion, that is, amylolysis. The two most important enzymes involved in starch hydrolysis are β‐amylase and α‐amylase, as listed in Table 2, along with limit dextrinase. Additionally, maltase, invertase, and α‐glucosidase contributed to the process during mashing, albeit to a lesser extent.

β‐amylase is an amylolytic exoenzyme and is already present in two forms, bound and free, in unmalted barley. Proteolytic enzymes make it possible to convert the bound form of the amylase into the free form (Narziß and Back 2019). It cleaves α‐1,4‐bonds of amylose and amylopectin starting from their non‐reducing ends. The degradation products are single maltose molecules whose reducing ends have a β‐configuration. Henson et al. (Henson and Duke 2016a; Henson et al. 2020) recently examined in detail the activity of β‐ and α‐amylase from two different malts in the presence of maltose and mannitol at 63°C, 68°C, 73°C, and 78°C and found that the stability of the enzymes depends on the wort composition. The activity of β‐amylase is highest at 63°C but rapidly loses activity. After 120 min, only 15% of the original activity was observed in the absence of maltose. However, in the presence of maltose, the enzyme is remarkably stable over 2 h at 63°C. At 73°C, the activity of β‐amylase decreases to about 10% of its original activity within 30 min, and the concentration of maltose plays only a minor role in the stability of the enzyme at this temperature. α‐Amylase, on the other hand, showed quite high activity at 73°C in the presence of maltose for at least 90 min. These data indicate that the activity of the two starch‐cleaving enzymes, β‐ and α‐amylase, is dependent on malt, temperature, and wort composition.

β‐amylase gradually decreases during a 60‐min rest period at 65°C, and its activity is approximately 40%–60% of its initial value at the end of this rest period. After the temperature is increased to 74°C, the enzyme ceases its activity completely (D. E. Evans 2017). C. F. De Schepper, Gielens, et al. (2021) show that the optimal activity temperature and inactivation temperature of β‐amylase are lower than previously assumed. Three activities can be described for β‐amylase, which is present in unmalted grains. One is the activity of free β‐amylase, which can be seen during malting and mashing, the potential activity of matrix‐bound β‐amylase, and the sum of these two activities. To achieve the highest possible diastatic power, protein‐bound amylases must be released during malting. Depending on the barley variety of the malt, the optimum temperature of β‐amylase varies in a temperature range between 45°C and 50°C. However, no starch gelatinization occurred at this temperature. The experiments were performed using soluble malt starch. Inactivation of β‐amylase starts at a temperature of 52.5°C. At a temperature of 60°C, only 10% of the activity remains after 3.4 min. The enzyme was completely deactivated after 8 min. In practical mashing experiments, the thermostability shifts because of the stabilizing effect of sugars on proteins. Low thermostability was also observed. Inactivation already starts during heating from 45°C to 62°C. At the beginning of the 62°C rest, β‐amylase is almost completely inactivated. However, a small residual activity of β‐amylase was also seen up to a temperature of 70°C for 60 min. Thus, the previously assumed optimum temperature of β‐amylase is impractical (Laus et al. 2022).

α‐Amylase in barley is formed from the second day of germination during malting (Bamforth 2009). The α‐amylase is an endoenzyme and hydrolyses from within the molecule. The molecules that can be attacked by α‐amylase are macromolecules of starch from the malt, which are composed of α‐1,4‐ or 1,6‐glycosidically bound glucose units. Here, the enzyme is able to cut α‐1,4 bonds inside the starch chains. Thus, larger starch chains or oligosaccharides with 6–7 glucose units and limited dextrins with α‐1,4‐ and 1,6‐bonds were released. α‐1,6 bonds are not cleaved. The action of α‐amylase releases new non‐reducing ends of starch chains and thus forms new targets for β‐amylase. On the basis of the action of α‐amylase, single glucose, maltose, and maltotriose molecules possibly form.

Although α‐amylase shows no decrease in activity for over 70 min at 65°C, its activity rapidly decreases to ∼20%–40% of its original activity after the temperature is increased to 74°C (D. E. Evans and Fox 2017). Henson and Duke (2016b) investigated α‐amylase activity at different temperatures and maltose concentrations. Irrespective of the maltose concentration in the mash, the activity of α‐amylase increases steadily at a temperature of 63°C for 30 min, after which it decreased slightly and then remains constantly active for another 60 min. After 30 min at 63°C, it reached its highest activity in almost all experiments performed. However, enzyme activity increased with increasing maltose concentration. At mash temperatures of 68°C, the activity curve is similar to the experiments at 63°C. The activity initially increased during the first 30 min and then dropped rapidly. Depending on the maltose content, a low residual activity of α‐amylase remains after 120 min of mashing. At elevated temperatures of 73°C and 78°C, the thermostability of α‐amylase is strongly dependent on the maltose concentration. Accordingly, maltose is assumed to have a protective function for α‐amylases at higher temperatures. The lower optimum and inactivation temperature already established for β‐amylase was also confirmed for α‐amylase by C. F. De Schepper et al. (2021). In dilute solutions of malt starch dissolved in water, the optimum temperature of α‐amylase is believed to be between a range of 55°C and 60°C. Inactivation in dilute solutions begins for the enzyme at a temperature of 60°C. In mashing experiments conducted in conditions similar to real ones, the inactivation of α‐amylase already begins at a temperature previously considered to be the optimal temperature of action, which is approximately 70°C. The optimal temperature in these experiments was at a temperature lower than 72°C. Therefore, in general, the resting temperatures during mashing can be assumed to be reduced in practice. Notably, the part of the α‐amylase was active until the end of mashing for 120 min at 80°C.

Limit dextrinase is able to cleave the α‐1,6‐bonds mainly found in amylopectin and, to a much lesser extent, in amylose. Thus, this enzyme is essential if the degree of fermentation of the wort reaches a high value by fermenting more sugars using yeast. Limit dextrinase is present in unmalted barley in bound or inactive forms.

In a recent study by D. E. Evans and Fox (2017), limit dextrinase shows a rapid increase in activity during the first 20 min of the 65°C rest and then decreases rapidly due to thermal deactivation. The activity initially increases by 50%–70% and then decreases to 45%–70% of its initial activity, which is maintained during the remainder of the resting period at 65°C. At 74°C, limit dextrinase activity decreases to between 15% and 30% of its initial value (D. E. Evans and Fox 2017).

### 3.2 New Developments in Mashing Systems

#### 3.2.1 Continuous Mashing

Development of a continuous brewing process is a major topic in brewing science. A continuous process promises more energy‐efficient operation, easier control, and thus more cost‐effective production (Meura 2008, 2013). This has already been implemented in some areas of beer production, such as malt milling, and can be easily developed. In other areas, such as fermentation, a continuous process is more difficult to implement but shows promising results with immobilized yeast (Mensour et al. 1997). In general, continuous systems already exist in the process steps upstream and downstream of mashing; these include the milling of malt or raw cereals, filtration of the mash using mash filters, boiling systems, hot trub separation, and cooling and aeration of wort (Willaert and Baron 2001; Kempfert 2016; Hertel and Sommer 2016).

Mashing is a step in which a continuous process has thus far only been established to a limited extent.

Mashing has always been a process that is carried out in batches. If this process step could be changed from a batch system to a continuous system, then advantages in terms of the specific product output, that is, an increase in the amount of product produced per unit space and time, as well as a smaller footprint, can be leveraged. The development of a continuous mashing process could contribute to more economical beer production (Strobl 2019). The advantages of continuous processes include smaller reactors, pumps, heat exchangers, valves, heat recovery, consistent energy consumption without peaks, and a reduced need for cleaning (Wefing et al. 2023). A major advantage of continuous mashing is the reduction in the total processing time. By utilizing the defined temperature profiles in separate reaction zones, enzymatic activities can be optimized for rapid and effective starch conversion. The continuous mode allows for precise control of process parameters such as temperature, residence time, and flow rate, resulting in consistent wort quality and minimization of variability. In a continuous system, enzymatic conversion processes in the mash can be controlled by the fill level, the flow rate (residence time), and the temperature (Wefing et al. 2020). Therefore, a continuous process can be flexibly adapted to change the raw material properties (Versteegh 1996). Continuous mashing offers a high degree of automation, which makes it particularly suitable for industrial‐scale breweries. When integrated into modern process control systems, real‐time monitoring and regulation can be achieved to ensure consistent operating conditions. In addition, the specific energy consumption may be reduced through the implementation of efficient heat recovery systems because the need for repeated heating and cooling phases is eliminated. Continuous mashing is less flexible in accommodating diverse mashing regimes required for specific beer styles, especially those relying on complex temperature profiles such as decoction mashing (Strobl 2019; Wefing et al. 2020).

Since the 1920s, attempts have been made to convert the mashing process from batch to continuous. Early systems included horizontal, vertical, plug‐flow, and continuously stirred tank reactors (CSTRs). However, the complexity of the mashing process has prevented satisfactory technical realization (Silhavy and Saginaw 1938; Huppmann 1969; Watts et al. 1964; Hudson and Button 1968; Kehse and Jess 1974; Moll et al. 1976; Mulder 2012; Mulder and Snip 2018; Versteegh 1996). As an example of an early system, Davis and Pollock (Davis and Pollock 1959) developed in 1958 a continuous mashing process in conjunction with the continuous extraction of wort. The milled malt was transferred to a conversion tube using a screw conveyor and mixed with water. It flows vertically downwards, is homogenized using an agitator, and reaches a pipe inclined at 30° at the bottom. At the lower end of the pipe, a disc filter is used to remove the first wort from the apparatus. The screw conveyor transports the remaining mash upward. It is fed into a vertical washing tube, in which the mash fell and reached another pipe inclined by 30°. Here, sparging water is fed into the system from above. It then flowed downwards and extracted the grist. Spent grains were transported upward by a screw conveyor, and the wort was removed from the process at the bottom via a disc filter. At the upper end of the second inclined pipe, the spent grains left the process. The entire mashing and lautering process takes place isothermally at 62°C and takes 30 min. However, the clarity of the lautered wort was poor, and it required further processing using a decanter. The values for available residual and soluble extracts were in the acceptable range of 0.5% (Davis and Pollock 1959).

Plant manufacturers GEA (Düsseldorf, Germany) and Meura have already developed mashing systems that represent a subprocess of integrated, continuous wort production (Schlenker 2019). Brewery 4.0 (GEA) is another system that realizes a continuous brewing process from mashing to filling. The continuous mashing process is based on a patent that expired in 1993 (Moll et al. 1976; Schlenker 2019). The pre‐mashed suspension passed through a reactor from the bottom to the top, which had three zones with different temperature ranges. Each of the three zones had its own heat‐medium circuit. The original invention uses water as the heating medium, which enters the heating pockets in the upper part of the zone and flows out again at the bottom. Brewery 4.0 uses steam injected directly into the mash, which means that no heating surfaces are required and fouling is avoided. The first zone has a temperature range of 35–50°C, the second a range of 50–65°C, and the third a range of 65–80°C. Temperatures can be adjusted according to needs and requirements owing to fluctuations in raw material quality. Each zone contained several condensed cells. These cells were arranged on top of each other, and each cell had its own agitator, which was mounted on a shaft that passed through the entire vertical reactor. In the individual Brewery 4.0, the agitators are designed as disc agitators. The mash is not subject to any rest period but flows continuously through the cells and the entire reactor. Three variants are recommended to ensure flow from bottom to top and prevent it from flowing back to the mash inlet:

I Propeller

The agitator was located centrally in the mash cell. A propeller was attached to the central shaft in the narrow space between the two cells. The rotating propeller produces a pumping effect in the vertical upward direction and prevents or reduces the flow of the mash back into the upstream mash cells.

II Valve

This backflow prevention method uses a valve that allows flow in only one direction. This valve can be designed as a disc around the central shaft, which is located at a narrow point between the two mash cells. The desired upward flow pushes the disc upward, and the mash can pass through the valve. If the liquid or particles press on the valve from above, they are pressed onto the constriction and backflow is prevented.

III Teardrop‐shaped cell

This mash cell design does not include a device to prevent the mash from flowing back. The agitator is located directly above the constriction between the two mash cells and has a low speed. The agitator generated a vertical centrifugal flow upward along the wall of the mash cell, and the flow into the upstream cells was reduced. Tear‐ or pear‐shaped cells proved to be advantageous in this case.

The agitator should not run too fast to avoid obstructing the upward flow. However, the stirring speed must not be too low to prevent the particles from settling. The settling of particles can clog the reactor and cause malfunctions in the system. The liquid phase of the mash flowed through the reactor faster than the particles. This allows for a stronger particle extraction (Schlenker 2019; Moll et al. 1976).

The Meurabrew system (Meura) was first used commercially in Belgium in 2007. As this process is based on mash filtration, a hammer mill, which can be operated continuously, was used to crush the malt. The mashing process was conducted in four separate tanks to enable continuous processing. These are a mashing tank at 45°C, two mashing tanks at 63°C and 72°C, and one mashing tank at 78°C. The temperature was kept constant, and the suspension had a certain resting time in each vessel. Vapor diffusion heats the vessel and prevents fouling. Meurabrew was able to cut water demand, energy, and steam consumption by 50%. The oxygen input into the mash and wort is lower, and a constant product quality could be achieved (Meura 2008, 2013).

**FIGURE 4:**
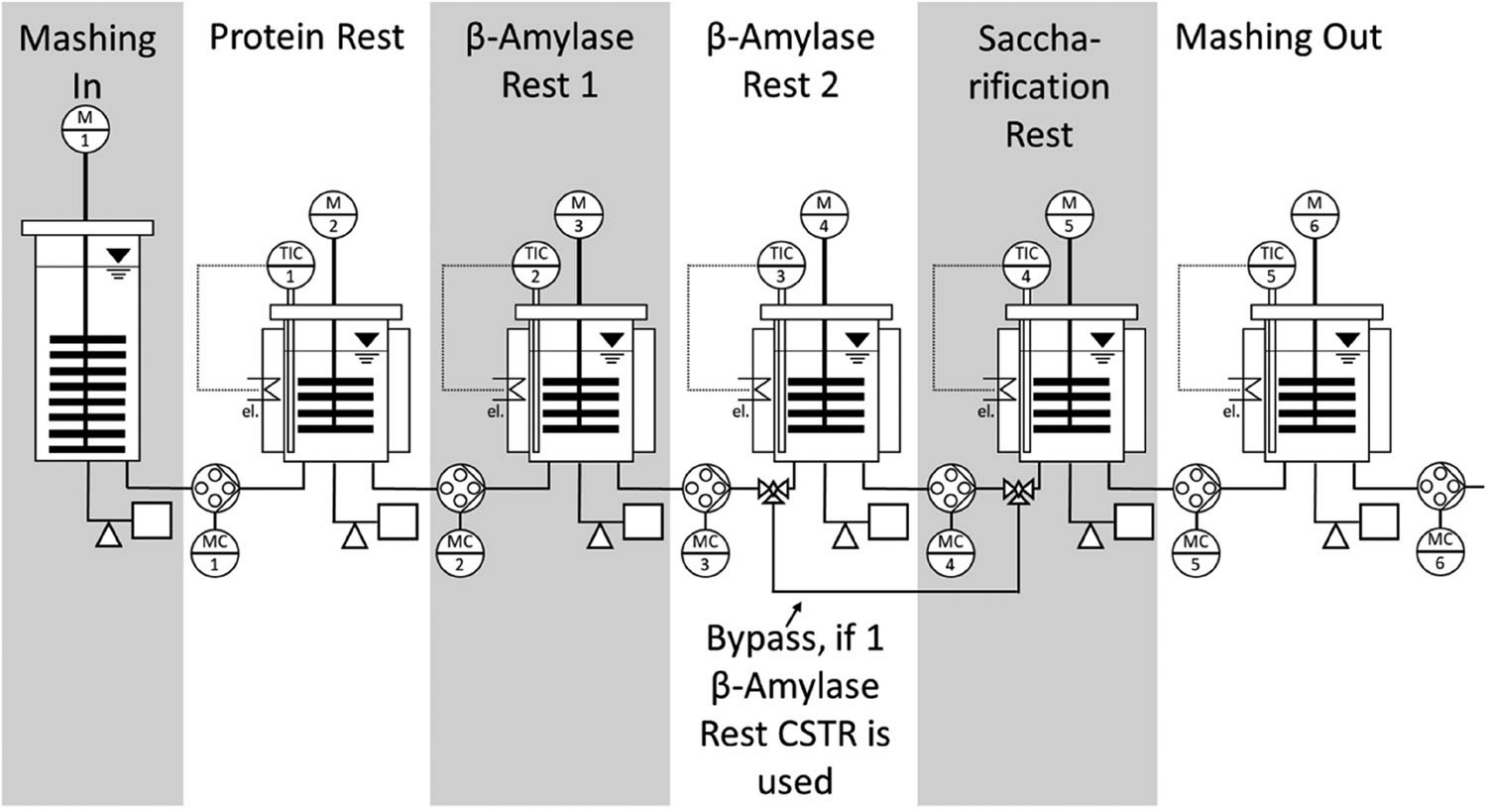
Experimental setup of a continuous mashing system with six reactors (Wefing et al. 2023).

Wefing et al. (2020) developed a continuous mashing system with the β‐amylase rest as the central point to achieve wort properties suitable for further processing. The majority of fermentable sugars are formed during the β‐amylase rest and are crucial for mash yield. The aim was to investigate a controlled β‐amylase rest in a CSTR. The amount of sugar formed during the β‐amylase rest depends on the temperature and the resting time. The CSTR pilot plant of this study consists of a single mashing reactor (2 L), a heating reactor (1 L), and one or optionally two stainless‐steel β‐amylase rest reactors (1 L net volume each) (Figure 4). Both the heating reactor (50°C) and the β‐amylase‐rest reactor (62–64°C) are equipped with an adjustable electric heating jacket. The reactors were connected using silicone hoses and peristaltic pumps. However, the informative value is linked to the malting and milling properties used and therefore cannot be transferred to the changed mashing conditions.

Newer studies have attempted to implement continuous brewing and mashing systems according to Industry 4.0. Industry 4.0, which is already an important field of research in many sectors, is also entering the brewing industry. After digitalization, which includes computerization and connectivity, the Industry 4.0 model has four basic components, namely, visibility, transparency, forecasting ability, and adaptability (Wefing et al. 2020; Schlenker 2019; Schuh et al. 2017). In a newer study, Wefing et al. (2020, 2023) have further developed a continuous mashing process with regard to Industry 4.0. A CSTR cascade was also used in this study (Wefing et al. 2023), which allowed a flexible and continuous mashing process. In this setup, the concentration of fermentable sugars formed was constantly measured during production and controlled by the mean residence time. Therefore, the residence time of the mash in the individual reactors was regulated by sugar content. A new type of continuous mashing system with a heterogeneous CSTR cascade was investigated, which enabled control of the extract, maltose, and glucose content. The construction of the continuous mashing system consisted of four steps:

I A protein rest, as well as cytolysis for β‐glucan degradation by β‐glucanase, which is important in poorly modified malts (Wefing et al. 2020). This is normally carried out at 48–52°C (Steiner et al. 2011) but can take place at 38°C (Jones and Marinac 2002; Jones 2005).
II A β‐amylase rest at 62–65°C (Hui 2007) to carry out amylolysis, the third phase.
III A saccharification rest, at 70–75°C (Hui 2007).
IV Mashing, as the last production step, which is carried out at 78°C.

The different temperature zones for the four mashing steps were considered independent but interconnected within the CSTRs. Either a single CSTR or a CSTR cascade consisting of two reactors was used for the β‐amylase rest. In this study, the mean residence time was used as a control parameter for the fermentable sugar content (maltose, glucose, and extract). The continuous mashing system consisted of seven stainless‐steel reactors with a mash‐in volume of 2 L in the CSTR, whereas all other CSTRs had a maximum volume of 1 L. The mash was then pumped using a peristaltic pump. The inner diameter of each hole was 4 mm. The flow rates were between 20 and 100 mL/min. The temperature settings of the CSTRs were determined using electric heating.

The system consists of a mashing vessel and six heatable vessels connected in series. Each held a volume of 1 L and was equipped with an oscillating agitator. The two vessels were then heated to the same temperature. The mash passes through the reactors and is exposed to different temperatures. Protein, maltose, and saccharification rests occur. This system allows the production of 144 L of mash per day. Using an inline near‐infrared (NIR) spectrometer, maltose content, apparent degree of attenuation, and extract content were determined in real time. Therefore, the system is at the visibility level of the maturity index for Industry 4.0. The aim is to continue this and integrate an adaptive control system so that the changing raw material quality and process characteristics can be automatically reacted to.

F. Zhang, Pinkal et al. (2019) further developed the above system to ensure consistent quality of the continuously produced wort using inline methods. This concept is based on closed‐loop controlled continuous mashing. The pilot plant is designed for the continuous production of mash with a flow rate of 0.3–6.0 L/h. A grist conveyor conveys the malt into a mashing vessel, and a series of downstream reactors are connected to each other. The reactor is regarded as a mash. In this approach, NIR and machine learning algorithms are applied for the online determination of sugar and free amino nitrogen (FAN), which are considered quality parameters, as the concentrations of sugar and FAN form the basis for the subsequent beer production process and, in the end, the flavor of the beer. The mash in the reactor was stirred using an oscillating agitator to ensure turbulent mixing. The retention time of the mash was controlled by adjusting the speed of the peristaltic pumps (variations in the flow rate and reactor level). The pumps are located between the reactors. Different residence times resulted in different sugar and FAN concentrations. An NIR inline probe monitors the maltose concentration of the mash during resting, whereas the data are analyzed in real time using machine learning algorithms to determine quality parameters such as maltose and FAN concentrations.

On the basis of cavitation with a Venturi nozzle, Albanese et al. (Meneguzzo 2017; Albanese et al. 2018) developed a pilot plant in which 12 hL of wort could be produced in a continuous process. This is described in detail in the subsequent section on cavitation.

#### 3.2.2 Cavitation

Cavitation is the name given to a process in which spontaneously formed bubbles or cavities implode in a short time, releasing enormous amounts of energy. This results in localized temperatures of between 727°C and 9727°C and pressures of between 100 and 5000 bar (Arya et al. 2023). Cavitation is caused by stress and energy dissipation in liquids. Different types of cavitation are distinguished depending on the manner in which spontaneous bubble formation is induced. Hydrodynamic and acoustic cavitations are the most important mechanisms in the food industry. In hydrodynamic cavitation, a pressure difference occurs because of the different flows in the liquids, causing bubbles to form. In acoustic cavitation, the pressure differences in liquids are caused by ultrasonic waves (Asaithambi et al. 2019).

Hydrodynamic cavitation, which is particularly suitable for processing liquid foodstuffs, can be caused by induced kinetic energy, for example, through the opening of a valve, a Venturi nozzle, or using high‐speed rotation devices. Typically, unintentional hydrodynamic cavitation occurs owing to the presence of high‐pressure pumps, turbines, and propellers (Arya et al. 2019; Castro‐Muñoz et al. 2023). The extraction of ingredients from plant materials is a growing field in which hydrodynamic cavitation is used. Hydrodynamic cavitation increases the extraction of polyphenols, flavonoids, and carotenoids and can be achieved by utilizing rotor–stator arrangements (Albanese and Meneguzzo 2019a).

**FIGURE 5:**
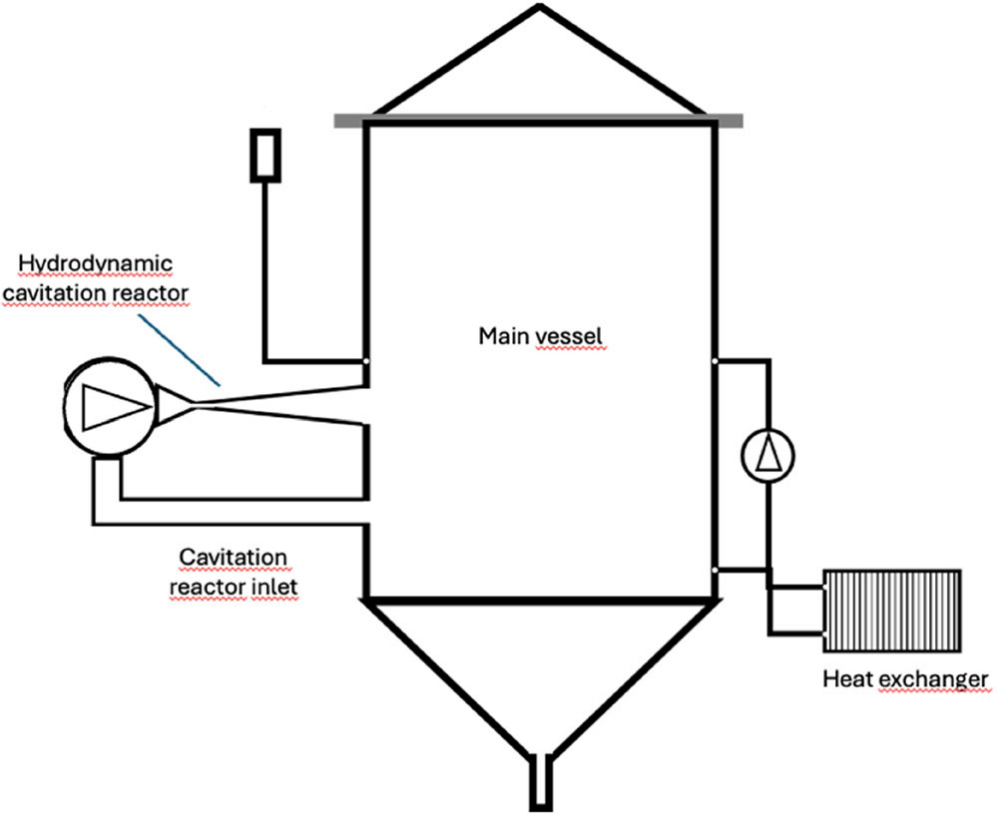
Experimental setup of a mashing device using hydrodynamic cavitation (Albanese et al. 2017).

Cavitation refers to the formation and subsequent decay of vapor bubbles. The higher the speed at which a liquid moves, the lower the hydrostatic pressure. On the moving parts of a fluid, this pressure can fall below the vaporization pressure of the liquid and bubbles can form. When the pressure increases again, these bubbles disintegrate and can damage the materials because of the pressure and temperature peaks. This effect can be used to crush and mash malt in water, as shown in the study by Albanese et al. (2015, 2016, 2017) and Albanese and Meneguzzo (2019b). The experimental setup shown in Figure 5 by Albanese et al. (2015, 2016, 2017) and Albanese and Meneguzzo (2019b) consists of a container with a capacity of 230 L and a centrifugal pump with which the mash is pumped into the circuit. The heat input into the liquid was achieved by converting the mechanical energy of the impeller of the pump into heat. The speed of the pump was set to 2300 rpm at a maximum pressure of approximately 4 bar and a maximum flow rate of 1500 L/min. To maintain rest and cool the wort, the system was equipped with a second smaller pump that conveyed the liquid to a plate heat exchanger. A cavitation reactor consisting of a Venturi nozzle and orifice plate was installed directly after the first pump. The tests were conducted using an unground malt and a malt grist. If grist was used, then it was placed in a container made of fine steel mash to ensure only the liquid was exposed to cavitation, and the influence on saccharification could be investigated. The tests were performed at atmospheric and higher pressures. The atmospheric pressure was sufficient to achieve satisfactory results.

Malt circulation through the cavitation reactor leads to pronounced comminution. Shear forces applied pulverize the grains to a size smaller than 100 µm. This also resulted in an increased starch extraction of 20%–30% and an increased starch concentration in the wort. A major advantage of this process is the low temperature required to achieve good enzymatic activity. With cavitation numbers in a range between 0.15 and 0.20, the temperature for activating enzymes fell by 35°C on average and the saccharification time could be reduced. This was due to the increased permeability of the cell membranes. At higher cavitation numbers, the activation temperature increases because of enzyme damage. The energy consumption of the brewhouse can be reduced by up to 30% compared to that of the conventional brewing process. In another study by Albanese et al. (2016), the gluten content of beer was reduced using hydrodynamic cavitation. Hydrodynamic cavitation assisted brewing eliminates the need for boiling, leading to a 34% reduction in electricity consumption (21 kWh/h compared to 32 kWh/h). Test comparisons showed a 33% energy savings, mainly due to the absence of the boiling stage and improved mashing efficiency (91% starch extraction vs. 60%). Additional advantages of hydrodynamic cavitation include the reduced or eliminated need for dry malt milling and cleaning, although milling is a low‐energy process (Albanese et al. 2017).

Cavibeer (Meneguzzo 2017) is a system with a size of 12 L/h that carries out the classic brewing process using hydrodynamic cavitation. The liquid was forced through a Venturi nozzle using a pump, which induced cavitation in the liquid. This enables various brewing processes to be performed. Owing to the high induced energy of cavitation, the raw material used (malt or unmalted cereals) does not need to be crushed and can be added directly to the water in the mash tube without being milled. Therefore, a milling unit was no longer necessary. The heat introduced by cavitation can be used to maintain different mashing rests, and the required wort boiling temperatures can also be achieved. This system is only powered by electricity, and time required and power consumption may be reduced by up to 50% compared to those of classic brewing processes. The specific applied energy of this system was approximately 10 kWh/h of beer.

With this system, it is possible to operate both in batch and continuous modes. A decanter is required to lauter the wort because the malt or unmalted cereal is crushed too finely for conventional lauter tuns, and the lauter tun clogs too quickly. During the batch process, mashing occurs in a tank and the wort is lautered through a decanter and stored in a buffer tank. Then, the wort was fed back into the cavitation tank for boiling and heated to the boiling temperature through the Venturi nozzle. In Cavibeer's continuous process, the buffer tank of the batch process is replaced by another cavitation tank, and the wort lautered by the decanter is fed into the tank for boiling (Meneguzzo 2017; Albanese et al. 2018; Meneguzzo and Albanese 2022).

Similar to a rotor–stator dispersion unit, a rotating pulsation device was used for mashing in a study by Safonova et al. (2019). The aim was to produce malt extract with reduced time expenditure. This system uses a rotor–stator unit. The Dispax mentioned rotor–stator system is used for crushing malt and mashing. In contrast, this system processes a water–malt grist suspension. Although the malt to be crushed is fed into the Dispax system from the rotor side, the mash in this process passes from the stator side to the inner working area of the tool. The rotor and stator are designed coaxially and have a variable number of teeth. Through the slots and three holes at the bottom of the rotor, the processed medium can reach the outer working area. Through a bypass, a mash that has not yet been fully processed can be returned to the system. The mashing was tested with variable process times, temperatures, and machine speeds. The machine works at a speed of up to 4000 rpm, and tests were carried out at mash temperatures between 60°C and 80°C. Because of the energy input through the rotor into the suspension, its temperature increases during processing, which must be taken into account to avoid exceeding the desired temperature. It has been shown that a speed of 2000 rpm at a mash temperature of 70°C achieved the best results. Complete saccharification of the starch was achieved after only 10 min. The beer produced from this wort was evaluated positively for tasting. Microcurrents that improve enzymatic processes are decisive for short processing times. Owing to the overlapping teeth of the rotor and stator, cavitation effects occur in these machines. As a result, dispersion is faster, and the resulting local temperature increase reduces the viscosity and has a positive effect on the diffusion of substances (Safonova et al. 2019, 2015).

#### 3.2.3 Combination of Milling and Mashing

In wet milling, crushing and mashing are combined. The malt is soaked in water at up to 50°C for 10–30 min or continuously sprayed with water at up to 70°C for ∼90 s and then crushed, usually using a two‐roller mill. This implied that the grist was already in the form of a mash. Mashing begins before grinding, and enzymatic activity begins at high temperatures (Back et al. 2008; Wronkowska 2016; Briggs et al. 2004). In general, the yield is the same for dry and wet milling, but the mashing time can be reduced for wet milling, or the concentration of reducing sugars can be increased for the same mashing time (de Moura and dos Santos Mathias 2018). One advantage of wet milling is the reduced lautering time because fermentable sugars are transferred more quickly from the filter bed to the sweet wort. Upon protecting the husks, fewer phenolic components are detected in the wort; however, the protein content increases (Szwajgier 2011).

Thus far, the combination of malt milling and mashing has only been used to a limited extent. Wet‐milling processes, such as those using two‐roller mills or newer milling systems, such as Dispax or T‐Rex systems, combine milling with mashing. However, the combination of a crusher and a mashing vessel in one container does not exist. This makes special mashing systems irrelevant, and the desired heating rate of the mash can be realized, if necessary, via the milling system used. A fundamental advantage of the combined system is its smaller space requirement. Grinding requires space in the brewhouse or on top of the mash tube. If the milling is performed inside a vessel, the warm area of the brewery can be designed to be more compact. The oxygen input, and thus the risk of oxidation of the mash, can be minimized by N_2_ or CO_2_ atmospheres because the grist does not have to be fed into the vessel, but malt can already be present. Similar to wet grinding and hydromill disc milling, where the rollers are underwater, the combined system is explosion‐proof. The input of heat with the help of the milling system must be examined more closely to determine whether heating the mash is energetically comparable to conventional methods or even more energy efficient.

## 4 Conclusions

New results corresponding to crushing processes, mashing and gelatinization, and the enzymatic properties of barley malt have been reported. When the malt is milled, attention must be paid to the desired degree of comminution. A very fine grind can lead to a higher amylose content and improve the enzymatic degradation during mashing because of lower unfermentable dextrins; however, excessive grindings may cause difficulties. A potential disadvantage of comminution is the leaching of undesirable substances from the malt components, primarily husks.

Recent studies have provided new insights into the gelatinization temperature of barley malt. In particular, small B‐type starch grains require high temperatures before they become vulnerable to enzymes. However, this temperature is strongly influenced by different factors. A high proportion of soluble substances in the malt increases the gelatinization temperature. Particularly, a high proportion of small starch grains leads to poor results during wort preparation.

Studies have shown that the optimal temperatures for malt enzymes have changed compared to previous observations.

Mashing can be optimized by adapting the resting temperatures to the enzymatic conditions of new grain varieties and cultivars. The mashing process can be shortened, or the energy required for mashing can be reduced by adjusting the temperature. It was found that the necessary amylases had lower inactivation temperatures than previously assumed. However, the thermal stability of the enzymes is also influenced by the presence of sugars in the mash. For example, an increased concentration of maltose leads to improved amylase stability, whereas a high concentration of proteins inhibits enzyme activity. In view of these conditions, the mashing processes should be adapted according to the gelatinization temperature and enzymatic activity of the malt. On one hand, resting temperatures must be maintained accordingly, and by using simplified mashing processes, such as isothermal mashing, the properties of the malt used can be easily accommodated.

A major issue in continuous beer production is not every stage is amenable to a continuous process. Although the crushing of malt or crude cereals is already a continuous process owing to the use of mills, the transformation of mashing into a continuous process is more challenging. Establishing a continuous mashing process is not particularly difficult when the space in the brewery is not of concern. However, owing to space requirements, using several vessels for the mashing step to maintain specific resting temperatures is not practical. The ability to combine milling and mashing could reduce the space requirement in the brewhouse because no additional comminution equipment would be necessary. In addition, in the case of rotor–stator devices, external heating of the mash tube is not required. Therefore, further research in the field of continuous beer production is required.

In practical trials, continuous mashing systems can, for example, have a cascade structure and maintain different resting temperatures in several mashing vessels, which can be adapted to the required temperatures. Long pipelines through which the mash is continuously fed can also pass through different resting temperatures in different heated zones.

Combining crushing and mashing in a single step can reduce space and energy requirements while maintaining or even improving yields. Dispersing machines or cavitation, which are used for the comminution of malt, as well as new technologies with regard to the heating and homogenization of mash, make the mashing step in beer production more energy‐efficient and increase the extract yield.

Hydrodynamic cavitation can crush malt directly in the mash tun, and the enzyme activity may be enhanced owing to hydrodynamic cavitation. Another possibility for combining crushing and mashing is the use of rotor–stator dispersing machines. Here, too, it would be possible to crush the malt directly in the mash tun using appropriate tools. However, to date, the production of wort extracts has only been tested using malt grist. The use of dispersing machines, hydrodynamic cavitation, and continuous mashing processes has significant potential.

Continuous process innovations, particularly the integration of the milling and mashing steps, offer significant opportunities to reduce energy consumption, save space, and enhance process efficiency. The application of technologies such as hydrodynamic cavitation and rotor–stator dispersing systems not only streamlines mash preparation but also improves enzymatic activity and extract yield. More detailed studies on the enzymatic behavior under cavitation and dispersal conditions are required to optimize the process parameters for different malt varieties.

Another crucial area is the development of real‐time monitoring and control systems that can dynamically adjust resting temperatures, flow rates, and enzyme activity, ensuring consistent quality even in continuous operations. By embracing continuous production concepts, the brewing industry can achieve higher sustainability targets, lower production costs, and open new possibilities for innovative beer styles and production flexibility.

## Author Contributions

**Andreas Laus**: conceptualization, investigation, writing – original draft, methodology. **Martin Zarnkow**: supervision, writing – review and editing, project administration. **Martina Gastl**: supervision, writing – review and editing, project administration. **Fritz Jacob**: supervision, writing – review and editing, project administration.

## Conflicts of Interest

The authors declare no conflicts of interest.
